# Insights into the biotic factors driving the outcome of coalescence events between soil bacterial communities

**DOI:** 10.1093/ismeco/ycaf048

**Published:** 2025-04-21

**Authors:** Sarah Huet, Sana Romdhane, Marie-Christine Breuil, David Bru, Arnaud Mounier, Laurent Philippot, Ayme Spor

**Affiliations:** University Bourgogne, INRAE, Institut Agro Dijon, Department of Agroecology, 17 rue de Sully, Dijon 21000, France; University Bourgogne, INRAE, Institut Agro Dijon, Department of Agroecology, 17 rue de Sully, Dijon 21000, France; University Bourgogne, INRAE, Institut Agro Dijon, Department of Agroecology, 17 rue de Sully, Dijon 21000, France; University Bourgogne, INRAE, Institut Agro Dijon, Department of Agroecology, 17 rue de Sully, Dijon 21000, France; University Bourgogne, INRAE, Institut Agro Dijon, Department of Agroecology, 17 rue de Sully, Dijon 21000, France; University Bourgogne, INRAE, Institut Agro Dijon, Department of Agroecology, 17 rue de Sully, Dijon 21000, France; University Bourgogne, INRAE, Institut Agro Dijon, Department of Agroecology, 17 rue de Sully, Dijon 21000, France

**Keywords:** microbial interactions, top–down manipulation, soil bacterial community, density-dependent interactions, coalescence

## Abstract

Coalescence events, which consist in the mixing of previously separated communities, are frequent in nature or as a result of human activities. Despite recently gaining attention as a tool to test ecological theories and engineer microbial communities, little is known about the factors that influence the outcome of such coalescence events. Here, we evaluated the relative importance of three community properties—namely, diversity, composition, and density—in determining coalescence outcome and biotic interactions among members of the coalescing bacterial communities. We found that manipulation of the density and composition of soil bacterial community resulted in the largest shifts in the structure of the resulting coalesced communities, explaining 24.7% and 6.8% of the variance in the β-diversity of the coalesced communities, respectively. Coalescence events impacted up to 35% of the dominant Operational Taxonomic Unit (OTUs) in the native community, with a predominance of negative effects. Our results also revealed that community density had the greatest explanatory power for the variance in the relative abundance of the OTUs negatively affected by coalescence events. In particular, all significantly affected OTUs that belonged to the Bacillales exhibited a decrease in relative abundance in several of the coalesced communities, which was related to the density of some members of the α-Proteobacteria and γ-Proteobacteria in the manipulated community suspensions. Overall, our data suggest that community density and composition were the main properties determining the outcome of coalescence events and that coalescence experiments can offer insights into multi-species interactions in complex environments.

## Introduction

Community coalescence, i.e. the encounter and interchange of entire communities, is a widespread phenomenon among microbes [1–3]. Microbial coalescence can result from various events, such as the mixing of soil and water [4], the application of organic fertilizers [5] or the excretion of feces [6]. Such mixing of previously separate microbial communities triggers a cascade of biotic interactions within and between the coalescing communities, leading to the formation of new assemblages with properties different from than the initial ones [7, 8]. Lately, coalescence received further attention in microbial ecology as a promising framework to understand the ecological processes determining the outcome of mixing events between different communities [3, 5, 8]. For example, Huet *et al*. combined removal and community coalescence experiments to assess the importance of biotic interactions in soil microbial community assembly [9]. The practical application of microbial coalescence as a tool to engineer microbial communities for optimized removal of contaminants in wastewater treatment systems or for the production of methane in industry also has been highlighted in a few studies [3, 8, 10]. While the success of coalescence-based strategies to manipulate microbial communities depends on the understanding of the underlying mechanisms, experimental studies exploring these mechanisms are lacking.

The outcome of community coalescence is largely determined by the biotic interactions that occur both during and after coalescence. These interactions are intrinsically linked to the diversity, composition, and density of the coalescing communities. For instance, more diverse communities are more likely to contain better competitors, increasing their chance to asymmetrically dominate over another after coalescence [8, 11]. A recent work by Liu & Salles [12] showed that the outcome of coalescence between bacterial communities could be predicted by similarity and unevenness indexes of compositional, functional, and metabolic traits. Several studies have also showed that a higher number of individuals increases the likelihood of their successful establishment in the coalesced community [11, 13–16], with this density effect being influenced by the composition of the resident community [11, 17]. Despite the importance of community diversity, composition and density in influencing biotic interactions occurring between invasive and resident microbial communities, knowledge of their combined effects in modulating the coalescence outcome is scarce.

**Figure 1:**
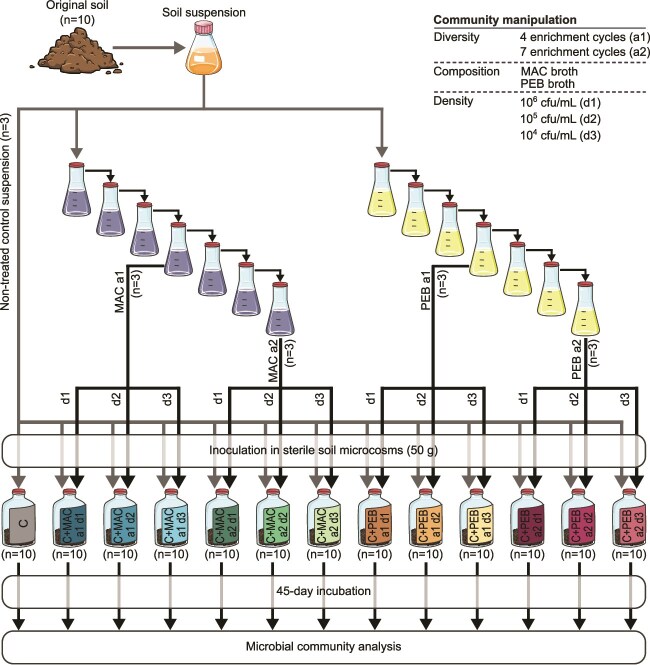
Schematic illustration of the experimental design. The soil microbial community was manipulated using a top-down approach. To manipulate microbial community composition, soil suspensions were subjected to a selective treatment consisting in an enrichment series in one out of two selective broths (i.e. MAC or PEB broth). Incubation series consisted in either 4 or 7 incubation rows to obtain two different diversity levels (a1 and a2, respectively). The same volume of the obtained manipulated suspensions was then diluted into increasing volumes of water to obtain three different densities (d1, d2, and d3). Coalescence events were performed by inoculating an equal mix of non-treated control suspensions and manipulated suspensions into sterile soil microcosms. The non-coalesced control community (C) was generated by inoculating and incubating the sole non-treated control suspension into sterile microcosms. Values in parentheses indicate the number of biological replicates.

Here, we sought to assess the relative importance of various microbial community properties in determining the outcome of coalescence events. For this purpose, we combined enrichment and dilution approaches to manipulate the diversity, composition and density (i.e. the total number of individuals) of a native soil microbial community. Then, each of the 12 resulting manipulated communities was mixed with the original community into microcosms containing the native sterilized soil (Fig. 1). We used linear and generalized linear mixed models along with co-occurrence network inference based on 16S rRNA metabarcoding data to evaluate the relative importance of the microbial diversity, composition and density in the coalescence outcomes and the underlying biotic interactions. By performing coalescence events between communities with different diversity, composition and density, our study provides new insights into the community properties that drive coalescence outcomes and biotic interactions in microbial communities.

## Material and methods

### Soil sampling and experimental design

A sandy loam soil (6.9% clay, 19% loam, and 74.1% sand, pH 5.5, and C and N content 14.7 g kg^–1^ and 1.19 g kg^–1^ dry soil, respectively) was collected at the research station CEREEP, France (48°16′59.5"N, 2°40′18.5"E) and sieved through 4 mm. Part of this soil was γ-sterilized (>60 kGy; Conservatome, Dagneux, France) to set up sterilized soil microcosms. The rest of the unsterilized soil was conserved at +4°C as the original soil. To produce communities with different compositions (Fig. 1), we applied two different selective treatments by inoculating 750 μl of a suspension of the original soil (dilution 1:100 in sterile water) into 15 ml of two different selective broths: Mac Conkey broth (MAC) and Phenylethyl alcohol broth (PEB). MAC is a selective and differential media that supports the growth of Gram-negative bacteria while inhibiting that of Gram-positive bacteria through the addition of bile salts. On the opposite, PEB is a selective media for Gram-positive bacteria, with phenylethyl alcohol inhibiting the growth of Gram-negative bacteria. Each incubation cycle lasts 3.5 days. At the end of each cycle, 300 μl of the incubated suspension was inoculated into 15 ml of fresh sterile broth. To obtain communities with different diversities, the suspensions were subjected to either 4 (a1) or 7 (a2) enrichment cycles before being washed three times as previously reported [18]. We checked that the four washed suspensions (i.e. MAC a1, MAC a2, PEB a1, and PEB a2) had similar densities ranging from 7.76e10^8^ to 1.84e10^9^ 16S rRNA gene copy number (Supplementary Table S1). To obtain manipulated suspensions with densities similar to the non-treated control suspension (i.e. 2.5 × 10^6^ gene copies per mL; Supplementary Table S1), we first diluted the enriched then washed manipulated suspensions to 1/200^th^ in sterile water resulting in the d1 density. This d1 density was then diluted either to 1/100^th^ or to 1/1000^th^ to obtain the d2 and d3 densities, respectively. Thereby, soil suspensions were subjected to two selective treatments (MAC and PEB), two enrichment cycle numbers (a1 and a2) and three dilutions (d1, d2, d3), resulting in twelve manipulated suspensions. Coalescence events were performed by mixing 75 ml of original soil suspension (i.e. non-treated control suspension) with 75 ml of each manipulated suspension. Subsequently, 13 ml of this mix was inoculated into a sterile microcosm containing 50 g of γ-sterilized soil (n = 10) to reach 80% of the water holding capacity. A non-coalesced control community was set by inoculating 13 ml of the non-treated control suspension into sterile microcosms (n = 10). Microcosms were closed with sterile lids allowing gas exchange and incubated for 45 days at 23°C, at soil moisture ranging between 60% and 80% of the soil water-holding capacity. The original soil, the washed suspensions and the soil from the microcosms were used for subsequent analyses.

### Assessment of microbial community composition and diversity

DNA was extracted from a total of 155 samples using the DNeasy PowerSoil-htp 96 well DNA isolation kit (Qiagen, France) according to the manufacturer’s instructions. To generate amplicons, a two-step PCR approach was used according to Berry *et al*. [19]. The V3-V4 hypervariable region of the 16S rRNA gene was amplified using the 341F (5′-CCTACGGGRSGCAGCAG-3′) and 805R (5′-GACTACCAGGGTATCTAAT-3′). The amplicon size was checked with 2% agarose gel and DNA concentration was estimated using the Quant-IT™ dsDNA HS Assay kit (Invitrogen™, Carlsbad, CA, USA). Final PCR products were purified and their concentration was normalized using the SequalPrep Normalization plate kit (Invitrogen™, Carlsbad, CA, USA). Sequencing was performed on MiSeq (Illumina, 2 × 250 bp) using the MiSeq reagent kit v2. Demultiplexing and trimming of Illumina adaptors and barcodes were done with Illumina MiSeq Reporter software (version 2.5.1.3). Sequence data from the 155 soil samples were analyzed using an in-house developed Python pipeline implemented in a Jupyter notebook (https://github.com/sarah-huet/BISCAL_xp2). Briefly, 16S rRNA gene sequences were assembled using PEAR [20] with default settings. Further quality checks were conducted using the QIIME pipeline [21] and short sequences were removed (<375 bp). Reference-based and de novo chimera detection, as well as OTUs clustering, were performed using VSEARCH [22] and the adequate reference databases (SILVA’s representative set of sequences; see [23]). The identity thresholds were set at 94% based on replicate sequencing of a bacterial mock community [24]. Representative sequences for each OTU were aligned using Infernal [25] and a phylogenetic tree was constructed using FastTree [26]. Taxonomy was assigned using UCLUST [27] and the SILVA database (138.1/2020) [23]. Nine samples with ˂8000 sequences were filtered out (n = 146).

### Quantification of microbial communities

The abundances of total bacterial communities were quantified by real-time quantitative PCR (qPCR) assays using 16S rRNA primers described by Muyzer *et al*. [28]. Real-time qPCR assays were carried out in a ViiA7 (Life Technologies, USA) with a Takyon Master Mix (Eurogentec, France) as previously described by Bru *et al*. [29]. Two independent runs were performed with a PCR efficiency of 98.58 and 99.29%. No template controls gave null or negligible values. PCR inhibitor presence was tested by mixing soil DNA extracts with either control plasmid DNA (pGEM-T Easy Vector, Promega, France) or water. No inhibition was detected in any case.

### Statistical analysis

Statistical analyses were conducted using R statistical software version 4.0.3 [30].

#### Statistical analysis of diversity metrics

Both α-diversity (i.e. observed species, Simpson’s reciprocal, Shannon and Faith’s Phylogenetic Diversity PD, see [31]) and β-diversity metrics (i.e. unweighted and weighted Unifrac distances between samples, see [32]) were calculated based on rarefied OTU table (8000 sequences per sample).

The α and β-diversity indices of the suspensions were first analyzed by using Welch’s t-tests and Bonferroni *P*-value correction to compare each suspension (n = 3) to the original soil (n = 10). Then, the manipulated suspensions were compared among each other using the same approach as for the coalesced communities described as follows (Equation 1).

To estimate the effect of each treatment on α-diversity metrics, we used ANOVAs based on a linear model (Equation 1) followed by Tukey's honestly significant difference (HSD) test (*P-*value ≤0.05) using the agricolae package (version 1.3–7) [33]. Normality and homogeneity of the residual distribution were verified. Considering that a diversity index $Y$, in any $j$ replicates of any $i$ treatment, follows a Gaussian distribution of mean $\overline{y}$ and variance ${\sigma}^2$ as $Y\sim N\left(\overline{y},{\sigma}^2\right)$, we used the following model:


(1) \begin{equation*} {Y}_{ij}=\mu +{\gamma}_i+{\varepsilon}_{ij},{\varepsilon_{ij}}_{1\le j\le 10}\ \text{iid}\sim N\left(0,{\sigma}^2\right) \end{equation*}

where $i=\left\{0,\dots, 12\right\}$ represents the non-coalesced control and the coalescence treatments, $j=\left\{1,\dots, 10\right\}$ represents the replicates, $\gamma$ is the fixed effect of the treatments and $\varepsilon$ is the model residuals. To estimate the effect of each treatment on β-diversity, we performed principal components analyses (PCoA) and permutational multivariate analysis of variance (PERMANOVA) using the ordin and adonis function of the vegan package (version 2.6–2), respectively [34], based on weighted Unifrac distance matrix. We also implemented pairwise comparisons between treatment using the pairwise.adonis function from the pairwise Adonis package (version 0.4).

To assess the relative contribution of the community properties to each α and β diversity metrics of the coalesced communities, we calculated the property F-values using ANOVAs based on a linear model (Equation 2). Normality and homogeneity of the residual distribution were verified. Considering that a diversity index $Y$, in any $j$ replicates of any coalescence treatment with $a$ diversity, $b$ composition and $d$ density, follows a Gaussian distribution of mean $\overline{y}$ and variance ${\sigma}^2$ as $Y\sim N\left(\overline{y},{\sigma}^2\right)$, we used the following model:


$$ {Y}_{ab d j}=\mu +{\alpha}_a+{\beta}_b+{\delta}_d+\left({\alpha \beta}_{ab}\right)+\left({\alpha \delta}_{ad}\right)+\left({\beta \delta}_{bd}\right)+\left({\alpha \beta \delta}_{ab d}\right)+{\varepsilon}_{ab d j}, $$

(2) \begin{equation*} {\varepsilon_{abdj}}_{1\le j\le 10}\ \text{iid}\sim N\left(0,{\sigma}^2\right) \end{equation*}

where $a=\left\{1,2\right\}$ represents the diversity, $b=\left\{1,2\right\}$ represents the composition, $d=\left\{1,2,3\right\}$ represents the density, $j=\left\{1,\dots, 10\right\}$ represents the replicates, $\alpha, \beta, \delta$ are the fixed effects of the diversity, composition and density of the manipulated communities, respectively, and their interactions, and $\varepsilon$ is the model residuals. For β diversity metric, we used the weighted Unifrac distances by calculating all pairwise distances between each replicate of the coalesced communities and each replicate of the non-coalesced control community.

#### Statistical analysis of OTUs

Statistical analyses of OTUs abundances were focused on the most abundant OTUs in microcosms. Briefly, low-abundance OTUs were filtered out of the count table by keeping OTUs that (i) represented >0.1% of the sequences in at least ten samples and (ii) were found in at least 60% of replicates for any given treatment, which resulted in 258 dominant OTUs. These dominant OTUs were used to build pruned trees using the ape package (version 5.7–1) [35] and were visualized using the Interactive Tree of Life (iTOL) webserver [36].

To estimate the effect of the community properties on each OTU abundance, we calculated the property F-values using ANOVAs based on the following generalized linear mixed model (Equation 3). Considering that an OTU of abundance $Y$, in any $j$ replicates of any coalescence treatment with $a$ diversity, $b$ composition and $d$ density, follows a Poisson distribution of parameter $\varLambda$ as $Y\sim P\left(\varLambda \right)$, we used the following model:


\begin{align*} {log}\left({\varLambda}_{ab d j}\right)=&{o}_{ab d j}+\mu +{\alpha}_a+{\beta}_b+{\delta}_d+\left({\alpha \beta}_{ab}\right)+\left({\alpha \delta}_{ad}\right)+\left({\beta \delta}_{bd}\right)\\&+\left({\alpha \beta \delta}_{ab d}\right)+{Z}_{ab d j}, \end{align*}

(3) \begin{equation*} {Z_{abdj}}_{1\le j\le 10}\ \text{iid}\sim N\left(0,{\sigma}^2\right) \end{equation*}

where $a=\left\{1,2\right\}$ represents the diversity, $b=\left\{1,2\right\}$ represents the composition, $d=\left\{1,2,3\right\}$ represents the density, $j=\left\{1,\dots, 10\right\}$ represents the replicates, $\alpha, \beta, \delta$ are the fixed effects of the diversity, composition and density of the manipulated communities, respectively, and their interactions, $o$ is the offset for each sample calculated as the log of the sample read sum and $Z$ is the random sampling effect modeling the data over-dispersion. The analysis was performed using the glmer function of the *lme4* package (version 1.1–27). Each model was tested against a null model (i.e. a model without the effect of the treatments) using likelihood-ratio test and *P-*value were corrected using a Bonferroni correction (adjusted Chi square *P-*value ≤0.05).

To estimate the effect of each treatment on each OTU abundance, we used a generalized linear mixed model. Considering that an OTU of abundance $Y$, in any $j$ replicates of any $i$ treatment, follows a Poisson distribution of parameter $\varLambda$ as $Y\sim P\left(\varLambda \right)$, we used the following model:


(4) \begin{equation*} {log}\left({\varLambda}_{ij}\right)={o}_{ij}+\mu +{\gamma}_i+{Z}_{ij},{Z_{ij}}_{1\le j\le 10}\ \text{iid}\sim N\left(0,{\sigma}^2\right) \end{equation*}

where $i=\left\{0,\dots, 12\right\}$ represents the non-coalesced control and the coalescence treatments, $j=\left\{1,\dots, 10\right\}$ represents the replicates, $\gamma$ is the fixed effect of the treatments, $o$ is the offset for each sample calculated as the log of the sample read sum and $Z$ is the random sampling effect modeling the data overdispersion. The analysis was performed using the glmer function of the *lme4* package (version 1.1–27). Each model was tested against a null model (i.e. a model without the effect of the treatments) using likelihood-ratio test and *P-*value were corrected using a Bonferroni correction (adjusted Chi square *P-*value ≤0.05). Subsequently, we implemented multiple pairwise comparisons on significative models with the emmeans function of the *emmeans* package (version 1.6.1) and *P-*values were corrected using a Bonferroni correction (*P-*value ≤0.05). A loglikelihood ratio test was applied when the OTU had a null abundance in one treatment and a median abundance higher or equal to 5 in the compared treatment (see code available online).

Phylogenetic signals were tested for the estimated effect of each coalescence treatment (Equation 4) and for the relative effect (F values) of the community properties (Equation 3) on each OTU abundance using the Pagel’s Lambda method of the phylosig function from the *phytools* package (version 1.5–1).

#### Inference of co-occurrence networks

To identify interacting OTUs and to explore to which extent the manipulated community properties influenced interactions between OTUs, co-occurrence networks were inferred across all microcosm samples (i.e. 130 samples) and based on the most abundant OTUs using the same filter as described above (i.e. 258 OTUs). Networks were inferred using a sparse multivariate Poisson log-normal (PLN) model with a latent Gaussian layer and an observed Poisson layer using the *PLNmodels* package [37] with an offset corresponding to the number of reads in each sample. The best network was selected using a Stability Approach to Regularization Selection (StARS) [38]. For visualization purposes, only partial correlations with |ρ| ≥ 0.06 were considered and were visualized using the Interactive Tree of Life (iTOL) webserver [36].

## Results

### Manipulation of the composition and diversity of the soil microbial community

**Figure 2:**
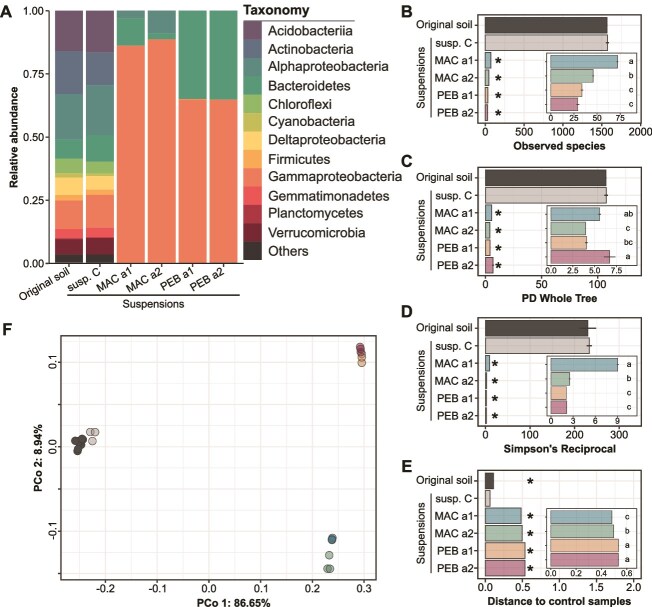
Composition, diversity and structure of the communities in the original soil, the non-treated control suspension and the manipulated suspensions. (A) Relative abundances of the fourteen most abundant class of bacteria in the original soil, the non-treated control suspension and the manipulated suspensions. (B–D) The number of observed species (B), the Faith’s phylogenetic diversity (C) and Simpson’s reciprocal (D) indices are shown (mean ± s.e.) in the original soil, the non-treated control suspension (C susp.) and the manipulated suspensions. Asterisks indicate suspensions significantly different than the original soil (Welch’s t-test *P-*value ≤0.05). (E) Weighted UniFrac distances between the non-treated control suspension samples and either themselves (susp. C) or the original soil samples or the manipulated suspension samples (mean ± s.e.). Asterisks indicate suspensions significantly different than the non-treated control suspension (Welch’s t-test *P-*value ≤0.05). (B–E) the insets focus on the manipulated suspensions (mean ± s.e.) where letters indicate significantly different statistical groups (Tukey’s test, *P-*value ≤0.05). (F) Principal coordinate analysis (PCoA) based on the weighted UniFrac distance matrix showing the original soil, the non-treated control suspension and the manipulated suspensions. The dot colors correspond to the bar colors in b to e.

To manipulate microbial diversity and composition, suspensions of the same homogenized soil were inoculated into MAC and PEB media. Successive enrichment cultures of 4 (a1) or 7 (a2) cycles (Fig. 1) resulted in a strong decrease in all α-diversity metrics with diversity losses ranging from 94% to 99% compared to the non-treated control suspension (Fig. 2A–D; Welch’s t-test *P-*value ≤0.05). The manipulated community MAC a1 was the most diverse, while the manipulated community PEB a2 had the lowest observed species and evenness but the highest phylogenetic diversity (i.e. PD whole tree) (Fig. 2B–D; Tukey’s HSD test, *P-*value ≤0.05). The composition of the manipulated communities was also significantly affected, with PEB a1 and PEB a2 being the most different from the non-treated control suspension (Fig. 2E and F; Tukey’s HSD test *P-*value ≤0.05). These shifts were characterized by a simplification of the community composition. Indeed, taxa belonging to α- and γ-Proteobacteria and Bacteroidetes represented 99.8% of the OTU relative abundance in manipulated suspensions (Fig. 2A). The bacterial abundance after the successive enrichment cultures in the MAC and PEB media ranged between 0.78 and 1.8 × 10^9^ 16S gene copies per ml (Supplementary Table S1). Subsequent dilution to 10^6^ (d1), 10^5^(d2), and 10^4^ (d3) gene copies per ml of the different washed cell suspensions were then performed to test the effects of all community properties (i.e. diversity, composition, and density) on the coalescence outcome when mixed with the non-treated control suspension. After incubating the inoculated microcosms for 45 days, the bacterial abundances observed across the various treatments were within the same range as the original soil (Supplementary Table S1), which indicates that the soil biotic capacity has been reached.

### Importance of microbial diversity, composition, and density for coalescence outcome

**Figure 3:**
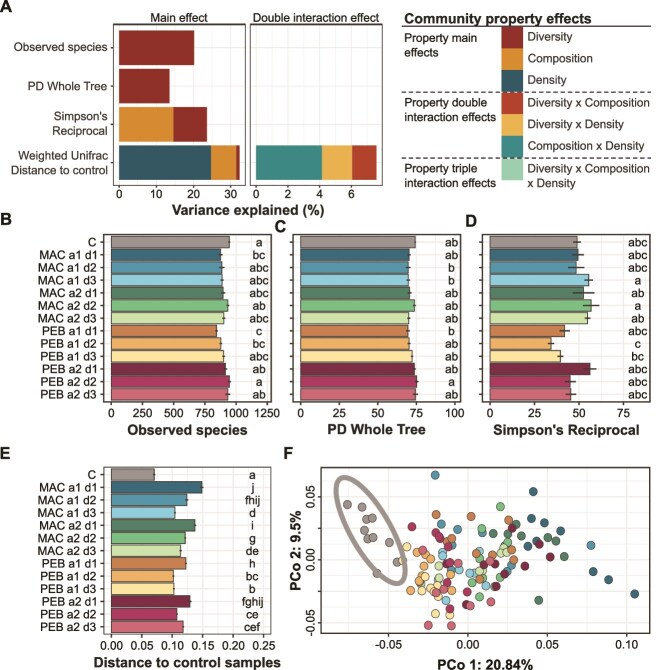
Community property and coalescence treatment effects on diversity and structure of the coalesced communities. (A) Bars show the percentage of variance explained by each community property on the diversity indices of the coalesced communities, as estimated using a linear model. (Bonferroni adjusted *P-*value ≤0.05). (B–D) the number of observed species (B), the Faith’s phylogenetic diversity (C) and Simpson’s reciprocal (D) indices are shown (mean ± s.e.) in the non-coalesced control and the coalesced communities. Letters indicate significantly different statistical groups (Tukey’s test, *P-*value ≤0.05). (E) Bars show the weighted UniFrac distances between the non-coalesced control samples and either themselves (C) or the coalesced communities (mean ± s.e.). Letters indicate significantly different statistical groups (Adonis pairwise comparison, Benjamini-Hochberg corrected *P-*value ≤0.05). (F) Principal coordinate analysis (PCoA) based on the weighted UniFrac distance matrix showing the non-coalesced control and the coalesced community samples and the 95% joint confidence ellipse for the non-coalesced control samples. The dot colors correspond to the bar colors in b to e.

To estimate the relative effect of each community property (i.e. diversity, composition, and density) on the diversity and composition of the coalesced communities, we used ANOVAs on linear models of the α and β-diversity indices (Equation 2). Manipulation of the microbial diversity explained part of the observed variance in the richness (20.1%) and in phylogenetic diversity (13.5%) of the coalesced communities while both microbial diversity and composition were important for the evenness of the coalesced communities with 9.0% and 14.6% of the variance in the Simpson’s Reciprocal index explained, respectively (Fig. 3A; Bonferroni adjusted *P-*value ≤0.05). In contrast, 24.7% of the variance in the β-diversity of the coalesced communities was explained by microbial density, 6.8% by composition, and only 0.9% by diversity (i.e. weighted Unifrac distance to non-coalesced control samples; Fig. 3A). None of the 2-way interactions were significant for the α-diversity metrics, whereas all 2-way interactions significantly explained a total of 7.6% of the variance in the β-diversity (Fig. 3A).

We then estimated how manipulation of microbial density, composition and diversity modulated the coalescence outcome by comparing the coalesced communities to the non-coalesced control community (non-treated control suspension incubated in microcosms without coalescence event; Fig. 1). Out of the twelve coalesced communities, three had a significantly lower richness than the non-coalesced control community (Fig. 3B and C; Tukey’s HSD test, *P-*value ≤0.05) with moderate losses of up to 10.8% of the observed species for the PEB a1d1 treatment. Significant differences in evenness were also observed between coalesced communities with Simpson’s Reciprocal indices of 34.3 and 56.8 in PEB a1d2 and MAC a2d2, respectively (Fig. 3D). When comparing weighted Unifrac distances between communities, the composition of all the coalesced communities was significantly different from the non-coalesced control and from each other in 78% of the pairwise comparisons (Supplementary Fig. S1; Fig. 3E and F; Supplementary Table S2; Adonis pairwise comparison, Benjamini-Hochberg corrected *P-*value ≤0.05). In particular, at the highest diversity (a1), the MAC community was more different from the non-coalesced control than the PEB community. In addition, for all coalesced communities, higher weighted Unifrac distances were observed between the non-coalesced control and the d1 coalesced communities than between the non-coalesced control and the d2 and/or d3 coalesced communities, which underlines the importance of community density in the coalescence outcome (Fig. 3E and F; Adonis pairwise comparison, Benjamini-Hochberg corrected *P-*value ≤0.05). Furthermore, when comparing unweighted Unifrac distances between communities, none of the coalesced communities were significantly different from the non-coalesced control, except PEB a1d1 (Supplementary Fig. S2; Adonis pairwise comparison, Benjamini-Hochberg corrected *P-*value ≤0.05). This suggests that the coalescence events primarily affected the relative abundance of the OTUs rather than their presence or absence.

### Identification of OTUs significantly impacted by coalescence

**Figure 4:**
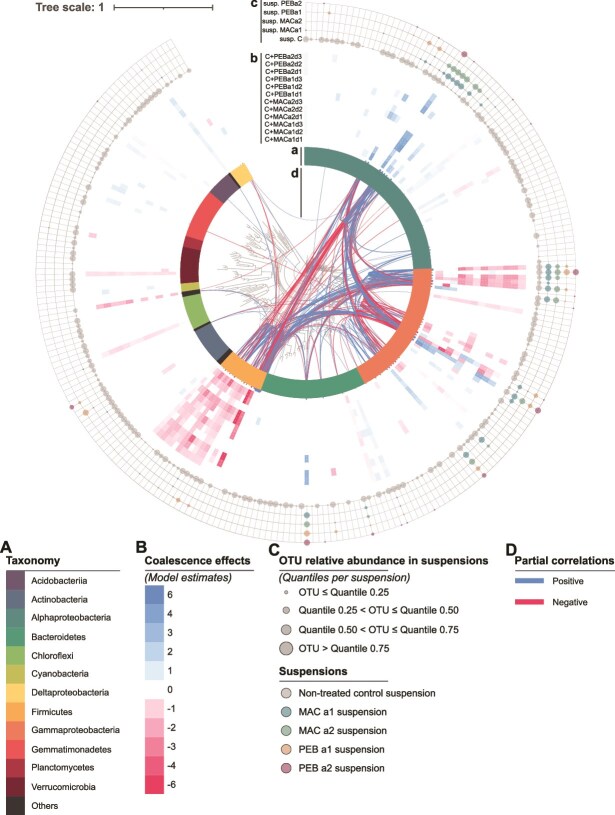
Taxonomic relationships and distribution of the 258 most abundant 16S rRNA OTUs. (A) The OTU class level is indicated by different colors on the innermost ring. (B) The inner rings show the coalescence treatment effects on the OTUs’ relative abundances compared to the non-coalesced control, as estimated using a generalized linear mixed model. The blue and red boxes indicate OTUs with increasing and decreasing relative abundance respectively, while white boxes indicate OTUs that are not affected by the treatment (Bonferroni adjusted *P-*value ≤0.05). (C) The outer ring shows the OTUs’ relative abundance in the non-treated control suspension and the manipulated suspensions. (D) The network, inferred from the non-coalesced control and the coalesced communities, is showed by the links displayed above the tree and representing partial correlations ρ colored blue if ρ > 0 and red if ρ < 0. (D).

To assess the relative effect of the community properties on OTU relative abundance, we performed an ANOVA on a generalized linear mixed model (Equation 3). Our results showed that manipulation of community diversity, composition, and density influenced the relative abundances of a total of 14.3%, 19.0%, and 22.1% of the dominant OTUs, respectively (Supplementary Fig. S3; Bonferroni adjusted *P*-value ≤0.05). Additionally, the different 2-way interactions influenced altogether 17.0% of the OTUs (Supplementary Fig. S3, Bonferroni adjusted *P-*value ≤0.05). Overall, 34.9% of the 258 most abundant OTUs exhibited significantly different relative abundances between coalesced and non-coalesced control communities (Fig. 4A and B; Bonferroni adjusted *P-*value ≤0.05). In all treatments but MAC a1 d3, more OTUs were negatively than positively impacted by the coalescence event (Supplementary Fig. S4a; Bonferroni adjusted *P*-value ≤0.05).

As expected, the number of impacted OTUs in the coalesced communities was the highest in the coalescence treatments performed with the manipulated communities at the highest density (d1; Supplementary Fig. S4a). However, the relative effect of community density was stronger for the negatively affected than for positively affected OTUs while the opposite was found for the effect of community composition (Supplementary Fig. S4b). The effects of community properties (Supplementary Fig. S3) and of the coalescence treatments (Fig. 4B) on the relative abundances of the dominant OTUs were significantly related to their taxonomic affiliation (Supplementary Table S3; Supplementary Fig. S5). For example, OTUs belonging to α-Proteobacteria were in overall positively affected by coalescence with a significant effect of community composition regardless of their relative abundance in the manipulated community suspensions (Fig. 4A–C). On the other hand, Firmicutes, which were abundant in the non-treated control suspension, were negatively affected, with a significant effect of both community density and composition (Fig. 4A–C, Supplementary Figs S3 and S5). Moreover, OTUs belonging to γ-Proteobacteria were also mostly negatively affected by coalescence with community diversity and density being the main drivers (Fig. 4A and B, Supplementary Figs S3 and S5). Interestingly, several OTUs belonging to the Pseudomonas sp within the γ-Proteobacteria, were negatively affected despite being abundant both in the non-treated control and manipulated community suspensions, therefore underlining the contribution of biotic interactions during coalescence.

### Coalescence Unraveled biotic interactions

To identify potentially interacting OTUs, we inferred a co-occurrence network using a sparse multivariate Poisson log-normal models across the coalesced and non-coalesced control communities (Fig. 4D). Among the 258 most abundant OTUs, 71 were connected by 88 negative and 142 positive links (Fig. 4D). Three phyla alone, α-Proteobacteria, Firmicutes, and γ-Proteobacteria, made up 83.1% of the network OTUs and 84.3% of all the network links (Fig. 4D). Notably, the highest number of negative links were between Rhizobiales (α-Proteobacteria) and Bacillales (Firmicutes) phyla (Supplementary Fig. S6). On the opposite, positive links were dominating between Bacillales and Pseudomonadales, β-Proteobacteriales or Xanthomonadales (Supplementary Fig. S6). Moreover, several of these OTUs belonging to the γ-Proteobacteria were also significantly associated to the α-Proteobacteria. No clear relationship was observed between the type of associations in the network (positive or negative) and the abundance of the corresponding OTUs in the suspensions.

## Discussion

Here, we used a top-down community manipulation approach to generate twelve microbial communities differing in their diversity, composition as well as density for assessing the relative importance of these community properties in the outcome of coalescence when mixed with a native soil community. In order to limit confounding factors arising from abiotic filtering and priority effects, all manipulated communities were generated using the same soil microbial community and were introduced simultaneously with the native community into their native sterilized soil. Another advantage of our approach is that it allows to test the extent to which a native soil microbial community can be steered through coalescence without introducing exogenous species.

We found that mixing separately the native soil community with 12 different manipulated communities resulted in coalesced communities exhibiting weak or no differences in α-diversity but large differences in their structure. This limited variability in the α-diversity metrics of the coalesced communities is likely due to the fact that all the manipulated communities had a much lower diversity that the native soil community. However, three coalescence events performed with manipulated communities having high densities (e.g. C + MACa1d1, C + PEBa1d1, and C + PEBa1d2) resulted in communities with lower richness than the non-coalesced control community. Since coalescence modifies the relative abundances of OTUs present in both communities, this lower richness can be explained by changes in density-dependent interactions, which have been shown to influence the likelihood of species coexistence [39]. Alternatively, our experimental design includes a range expansion phase that occurred during the microcosm colonization and that could affect the competition-colonization trade-off, consequently also influencing the diversity of the coalesced communities [39–41]. The manipulation of community composition using the MAC and PEB media led to significantly different bacterial communities, although differences between the two media were smaller than expected due the growth of several Gammaproteobacteria in the PEB media. Nevertheless, these changes in the composition but also in the density of the manipulated communities had the strongest effect on the structure of the coalesced community, as measured by weighted UniFrac distance to the non-coalesced control. These results are consistent with previous studies showing that community composition plays a key role in coalescence events [12, 16]. Such effect of the community composition has been partly attributed to phylogenetic similarity between invaders and resident communities, which could either hinder or increase the success of invasion [12, 15]. However, in our study the strong effect of the manipulation of community composition on the coalescence outcome cannot be explained by the phylogenetic similarity alone since all members of the manipulated communities were also present in the non-coalesced control community although at different densities.

Since the largest shifts in the composition of the coalesced communities were observed when coalescence was performed using the manipulated community suspensions with the highest density (d1), our results also highlight the role of density-dependent interactions in coalescence events. These findings are consistent with studies indicating that higher densities of invading cells result in more important changes in the native community structure [11, 13–16]. As manipulating the community density using dilution approaches can also cause the loss of rare species [42, 43], we cannot rule out that such density effect might also result from putative changes in community composition and diversity. However, since the diversity of the manipulated MAC and PEB communities was already very low at high density, it is likely that the number of rare species lost by dilution was limited.

The differential abundance analysis revealed that despite the low richness of the manipulated community suspensions, 35% of the most abundant OTUs in the non-coalesced control community were affected by the coalescence treatments. Additionally, the number of OTUs exhibiting decreased relative abundances was more than two times higher than that of those with increased relative abundances (Fig. 4B), which suggests that the coalescence treatments triggered negative rather than positive interactions. In line with our findings, previous work highlighted the importance of negative interactions in microbial community assembly using pairwise species mixture or top-down community manipulation [9, 44]. A recent study by Liu & Salles [12] even suggested that coalescence is a lose-lose game for both invader and resident community members. We also found that community density had the greatest explanatory power of the variance in the relative abundance of the negatively affected OTUs only. This indicates that harmful effects may have a stronger density dependence than beneficial ones during coalescence, potentially fostering coalesced community stability [39]. Double and triple interaction effects also explained significant proportions of the variation in the OTUs relative abundance, further supporting that the community properties can modulate each other’s effects on the likelihood of successful establishment [11, 17].

We found that all dominant OTUs belonging to the Bacillales within the Firmicutes phyla exhibited a decrease in relative abundance in several of the coalesced communities. This consistent negative impact of coalescence events on Bacillales was related to the density and to the composition of the manipulated community suspension as well as their interactive effects. Accordingly, we found that Bacillales were mostly associated by negative links to OTUs belonging to the α-Proteobacteria as previously reported [9, 24]. This suggests that Bacillales were detrimentally affected by members of the α-Proteobacteria when the latter were abundant in the manipulated community suspension. However, shifts in the relative abundances of Bacillales in the coalesced communities were not always mirrored in those of the α-Proteobacteria, which suggests that higher order rather than first order interactions are contributing to the outcome of coalescence. Thus, Bacillus were also associated by positive links to several y-Proteobacteria OTUs that were abundant in the manipulated communities. The decrease in relative abundance of the OTUs belonging to these two taxa after coalescence supports a recent work by Li and Salles [12] describing a lose-lose situation where both invaders and residents were negatively affected by coalescence.

In conclusion, our results unravel the differential importance of bacterial community composition, density and diversity in the outcome of coalescence events. In overall, community density and, to a lesser extent, community composition, were the strongest drivers of the structure of the coalesced communities. However, we also observed large differences in the contribution of the different community properties to the shifts in the relative abundances of the dominant OTUs, which were related to their taxonomy affiliation. The decrease in relative abundance of Bacillales when Proteobacteria were more abundant in the manipulated community suspensions has major implications, as it provides evidence for bacterial community assembly rules that can be used to predict how certain species will fare within the coalescing communities. Finally, we showed that coalescence experiments can offer insights into multi-species interactions in complex environments, which is invaluable for eventually engineering complex microbial communities for our benefit.

## Supplementary Material

HUET_ISME_Com_supp_mat_revised_20250311_ycaf048

## Data Availability

Raw 16S rRNA sequences were deposited at the NCBI under the accession number PRJNA1098960. All code and data are available on gitlab at the following link: https://gitlab.com/micro_bio_info_sarah/huet_2022.
