# Supplementary material for: Insights into the biotic factors driving the outcome of coalescence events between soil bacterial communities
Source: ISME Commun. 2025 Apr 21;5(1):ycaf048. doi: 10.1093/ismeco/ycaf048 (PMC12011082; doi:10.1093/ismeco/ycaf048)
Supplement: HUET_ISME_Com_supp_mat_revised_20250311_ycaf048 [file huet_isme_com_supp_mat_revised_20250311_ycaf048.pdf]

Supplementary Material

Supplementary Tables

Supplementary Table 1: Abundances of total bacteria. Quantification of 16S rRNA gene copy numbers in the original soil, the manipulated and inoculated suspensions, and microcosm soils (mean ± s.e. expressed as gene copy ml-1 suspension or g-1 dry soil).

Treatment	16S rRNA gene copy numbers	
	Mean	S.E.
Original Soil	2.49E+08	7.32E+06
Non-treated control suspension	2.55E+06	1.53E+05
MAC a1 suspension	1.23E+09	2.56E+07
MAC a2 suspension	7.76E+08	3.11E+08
PEB a1 suspension	1.84E+09	3.16E+07
PEB a2 suspension	1.57E+09	1.97E+08
Non-coalesced control	1.94E+08	1.31E+07
C + MAC a1 d1	2.27E+08	5.07E+06
C + MAC a1 d2	2.72E+08	2.17E+07
C + MAC a1 d3	2.36E+08	9.00E+06
C + MAC a2 d1	2.32E+08	8.29E+06
C + MAC a2 d2	1.95E+08	8.51E+06
C + MAC a2 d3	2.13E+08	4.56E+06
C + PEB a1 d1	1.98E+08	8.36E+06
C + PEB a1 d2	2.29E+08	1.61E+07
C + PEB a1 d3	2.59E+08	1.31E+07
C + PEB a2 d1	2.30E+08	1.33E+07
C + PEB a2 d2	2.37E+08	7.03E+06
C + PEB a2 d3	2.36E+08	3.95E+06

Supplementary Table S2: Pairwise comparison of weighted Unifrac distances between the non-control and coalesced communities.

Comparisons	Df	Sums Of Squares	F-values	R2	Adjusted p-value (Benjamini-Hochberg)	
C vs MAC_a1d1	1	5.90E-02	1.67E+01	4.95E-01	1.81E-03	*
C vs MAC_a1d2	1	4.32E-02	7.95E+00	3.06E-01	6.37E-03	*
C vs MAC_a1d3	1	9.91E-02	4.83E+01	7.40E-01	1.81E-03	*
C vs MAC_a2d1	1	6.24E-02	2.41E+01	6.01E-01	1.81E-03	*
C vs MAC_a2d2	1	9.56E-02	5.17E+01	7.52E-01	1.81E-03	*
C vs MAC_a2d3	1	9.31E-02	4.01E+01	7.15E-01	1.81E-03	*
C vs PEB_a1d1	1	4.83E-02	2.32E+01	5.63E-01	1.81E-03	*
C vs PEB_a1d2	1	7.18E-02	2.84E+01	6.12E-01	1.81E-03	*
C vs PEB_a1d3	1	7.46E-02	4.23E+01	7.02E-01	1.81E-03	*
C vs PEB_a2d1	1	6.35E-02	1.04E+01	3.67E-01	1.81E-03	*
C vs PEB_a2d2	1	8.42E-02	3.67E+01	6.83E-01	1.81E-03	*
C vs PEB_a2d3	1	5.92E-02	2.21E+01	5.65E-01	1.81E-03	*
MAC_a1d1 vs MAC_a1d2	1	2.98E-02	4.14E+00	1.96E-01	5.08E-02	
MAC_a1d1 vs MAC_a1d3	1	1.03E-01	2.77E+01	6.34E-01	1.81E-03	*
MAC_a1d1 vs MAC_a2d1	1	1.76E-02	4.00E+00	2.11E-01	4.03E-02	.
MAC_a1d1 vs MAC_a2d2	1	7.05E-02	2.02E+01	5.58E-01	1.81E-03	*
MAC_a1d1 vs MAC_a2d3	1	8.67E-02	2.11E+01	5.84E-01	1.81E-03	*
MAC_a1d1 vs PEB_a1d1	1	3.95E-02	1.09E+01	3.90E-01	1.81E-03	*
MAC_a1d1 vs PEB_a1d2	1	1.03E-01	2.50E+01	5.95E-01	1.81E-03	*
MAC_a1d1 vs PEB_a1d3	1	1.06E-01	3.21E+01	6.53E-01	1.81E-03	*
MAC_a1d1 vs PEB_a2d1	1	3.60E-02	4.57E+00	2.12E-01	5.03E-02	
MAC_a1d1 vs PEB_a2d2	1	9.68E-02	2.44E+01	6.04E-01	1.81E-03	*
MAC_a1d1 vs PEB_a2d3	1	6.64E-02	1.52E+01	4.87E-01	1.81E-03	*
MAC_a1d2 vs MAC_a1d3	1	2.97E-02	5.21E+00	2.35E-01	6.37E-03	*
MAC_a1d2 vs MAC_a2d1	1	9.78E-03	1.51E+00	8.63E-02	2.31E-01	
MAC_a1d2 vs MAC_a2d2	1	1.74E-02	3.16E+00	1.57E-01	3.97E-02	.
MAC_a1d2 vs MAC_a2d3	1	2.27E-02	3.66E+00	1.86E-01	3.97E-02	.
MAC_a1d2 vs PEB_a1d1	1	1.16E-02	2.10E+00	1.04E-01	1.25E-01	
MAC_a1d2 vs PEB_a1d2	1	2.99E-02	5.00E+00	2.17E-01	1.80E-02	.
MAC_a1d2 vs PEB_a1d3	1	2.94E-02	5.65E+00	2.39E-01	1.81E-03	*
MAC_a1d2 vs PEB_a2d1	1	6.19E-03	6.49E-01	3.48E-02	4.65E-01	
MAC_a1d2 vs PEB_a2d2	1	2.39E-02	4.02E+00	1.91E-01	1.80E-02	.
MAC_a1d2 vs PEB_a2d3	1	1.19E-02	1.87E+00	9.92E-02	1.73E-01	
MAC_a1d3 vs MAC_a2d1	1	4.27E-02	1.57E+01	5.12E-01	1.81E-03	*
MAC_a1d3 vs MAC_a2d2	1	1.21E-02	6.30E+00	2.82E-01	1.81E-03	*
MAC_a1d3 vs MAC_a2d3	1	3.12E-03	1.28E+00	7.88E-02	2.49E-01	
MAC_a1d3 vs PEB_a1d1	1	3.53E-02	1.63E+01	4.90E-01	1.81E-03	*
MAC_a1d3 vs PEB_a1d2	1	1.20E-02	4.57E+00	2.12E-01	3.32E-03	*
MAC_a1d3 vs PEB_a1d3	1	9.88E-03	5.42E+00	2.42E-01	1.81E-03	*
MAC_a1d3 vs PEB_a2d1	1	2.52E-02	3.95E+00	1.88E-01	3.32E-03	*
MAC_a1d3 vs PEB_a2d2	1	6.33E-03	2.64E+00	1.42E-01	4.55E-02	.
MAC_a1d3 vs PEB_a2d3	1	1.11E-02	3.96E+00	1.98E-01	2.98E-02	.
MAC_a2d1 vs MAC_a2d2	1	1.99E-02	8.03E+00	3.49E-01	1.81E-03	*
MAC_a2d1 vs MAC_a2d3	1	3.10E-02	1.01E+01	4.19E-01	1.81E-03	*
MAC_a2d1 vs PEB_a1d1	1	1.55E-02	5.74E+00	2.64E-01	1.81E-03	*
MAC_a2d1 vs PEB_a1d2	1	5.19E-02	1.62E+01	5.03E-01	1.81E-03	*
MAC_a2d1 vs PEB_a1d3	1	5.36E-02	2.29E+01	5.88E-01	1.81E-03	*
MAC_a2d1 vs PEB_a2d1	1	7.90E-03	1.10E+00	6.42E-02	3.47E-01	

MAC_a2d1 vs PEB_a2d2	1	4.11E-02	1.37E+01	4.78E-01	1.81E-03	*
MAC_a2d1 vs PEB_a2d3	1	2.66E-02	7.78E+00	3.41E-01	3.32E-03	*
MAC_a2d2 vs MAC_a2d3	1	5.03E-03	2.29E+00	1.32E-01	4.60E-02	.
MAC_a2d2 vs PEB_a1d1	1	2.88E-02	1.48E+01	4.65E-01	1.81E-03	*
MAC_a2d2 vs PEB_a1d2	1	2.98E-02	1.23E+01	4.19E-01	1.81E-03	*
MAC_a2d2 vs PEB_a1d3	1	2.55E-02	1.57E+01	4.81E-01	1.81E-03	*
MAC_a2d2 vs PEB_a2d1	1	1.20E-02	1.93E+00	1.02E-01	1.04E-01	
MAC_a2d2 vs PEB_a2d2	1	1.23E-02	5.67E+00	2.61E-01	1.81E-03	*
MAC_a2d2 vs PEB_a2d3	1	1.17E-02	4.54E+00	2.21E-01	1.81E-03	*
MAC_a2d3 vs PEB_a2d2	1	6.42E-03	2.37E+00	1.37E-01	8.26E-02	
PEB_a1d1 vs MAC_a2d3	1	2.65E-02	1.09E+01	4.05E-01	1.81E-03	*
PEB_a1d1 vs PEB_a1d2	1	2.60E-02	9.92E+00	3.55E-01	1.81E-03	*
PEB_a1d1 vs PEB_a1d3	1	3.47E-02	1.87E+01	5.09E-01	1.81E-03	*
PEB_a1d1 vs PEB_a2d1	1	1.13E-02	1.83E+00	9.23E-02	1.35E-01	
PEB_a1d1 vs PEB_a2d2	1	2.88E-02	1.20E+01	4.14E-01	1.81E-03	*
PEB_a1d1 vs PEB_a2d3	1	1.74E-02	6.25E+00	2.69E-01	1.81E-03	*
PEB_a1d2 vs MAC_a2d3	1	1.68E-02	5.71E+00	2.63E-01	1.81E-03	*
PEB_a1d2 vs PEB_a1d3	1	3.26E-03	1.41E+00	7.27E-02	2.49E-01	
PEB_a1d2 vs PEB_a2d1	1	3.15E-02	4.76E+00	2.09E-01	3.32E-03	*
PEB_a1d2 vs PEB_a2d2	1	8.42E-03	2.93E+00	1.47E-01	5.12E-02	
PEB_a1d2 vs PEB_a2d3	1	9.89E-03	3.03E+00	1.51E-01	6.17E-02	
PEB_a1d3 vs MAC_a2d3	1	1.50E-02	7.22E+00	3.11E-01	1.81E-03	*
PEB_a1d3 vs PEB_a2d1	1	3.18E-02	5.43E+00	2.32E-01	1.81E-03	*
PEB_a1d3 vs PEB_a2d2	1	6.32E-03	3.06E+00	1.53E-01	2.60E-02	.
PEB_a1d3 vs PEB_a2d3	1	7.62E-03	3.11E+00	1.55E-01	2.21E-02	.
PEB_a2d1 vs MAC_a2d3	1	1.65E-02	2.38E+00	1.30E-01	4.23E-02	.
PEB_a2d1 vs PEB_a2d2	1	2.37E-02	3.57E+00	1.74E-01	9.36E-03	*
PEB_a2d1 vs PEB_a2d3	1	9.99E-03	1.42E+00	7.72E-02	2.54E-01	
PEB_a2d3 vs MAC_a2d3	1	8.88E-03	2.83E+00	1.59E-01	5.55E-02	
PEB_a2d3 vs PEB_a2d2	1	8.01E-03	2.62E+00	1.41E-01	5.55E-02	

Supplementary Table 3: Phylogenetic signal (Pagel's lambda) tested for the estimated effect of each coalescence treatment and for the relative effect (F values) of the community properties.

	Pagel's Lambda	Loglikelihood Ratio	Chi^2 test p-value	Bonferroni adjusted p-value	
Community Properties					
Diversity	0.895425	22.76889	1.83E-06	1.28E-05	***
Composition	0.952783	77.23639	1.52E-18	1.06E-17	***
Density	0.328843	32.52335	1.18E-08	8.24E-08	***
Diversity x Composition	0.863811	60.21223	8.52E-15	5.96E-14	***
Diversity x Density	6.61E-05	-0.00678	1	7	
Composition x Density	0.793861	23.46168	1.27E-06	8.92E-06	***
Diversity x Composition x Density	0.867409	3.445148	0.063438	0.444064	
Coalescence treatments					
MAC a1 d1	0.560454	62.79929	2.29E-15	2.75E-14	***
MAC a1 d2	0.250464	18.99243	1.31E-05	0.000157	***
MAC a1 d3	0.032174	1.184835	0.276374	3.316486	
MAC a2 d1	0.628975	58.88712	1.67E-14	2.00E-13	***
MAC a2 d2	0.75636	53.07266	3.21E-13	3.86E-12	***
MAC a2 d3	0.955069	54.43757	1.60E-13	1.93E-12	***
PEB a1 d1	0.451438	44.512	2.53E-11	3.03E-10	***
PEB a1 d2	0.197626	9.419926	0.002146	0.025757	*
PEB a1 d3	0.420492	29.46757	5.69E-08	6.82E-07	***
PEB a2 d1	0.325875	24.68018	6.77E-07	8.12E-06	***
PEB a2 d2	0.357531	20.52098	5.90E-06	7.08E-05	***
PEB a2 d3	0.811101	34.71015	3.83E-09	4.59E-08	***

Supplementary Figures

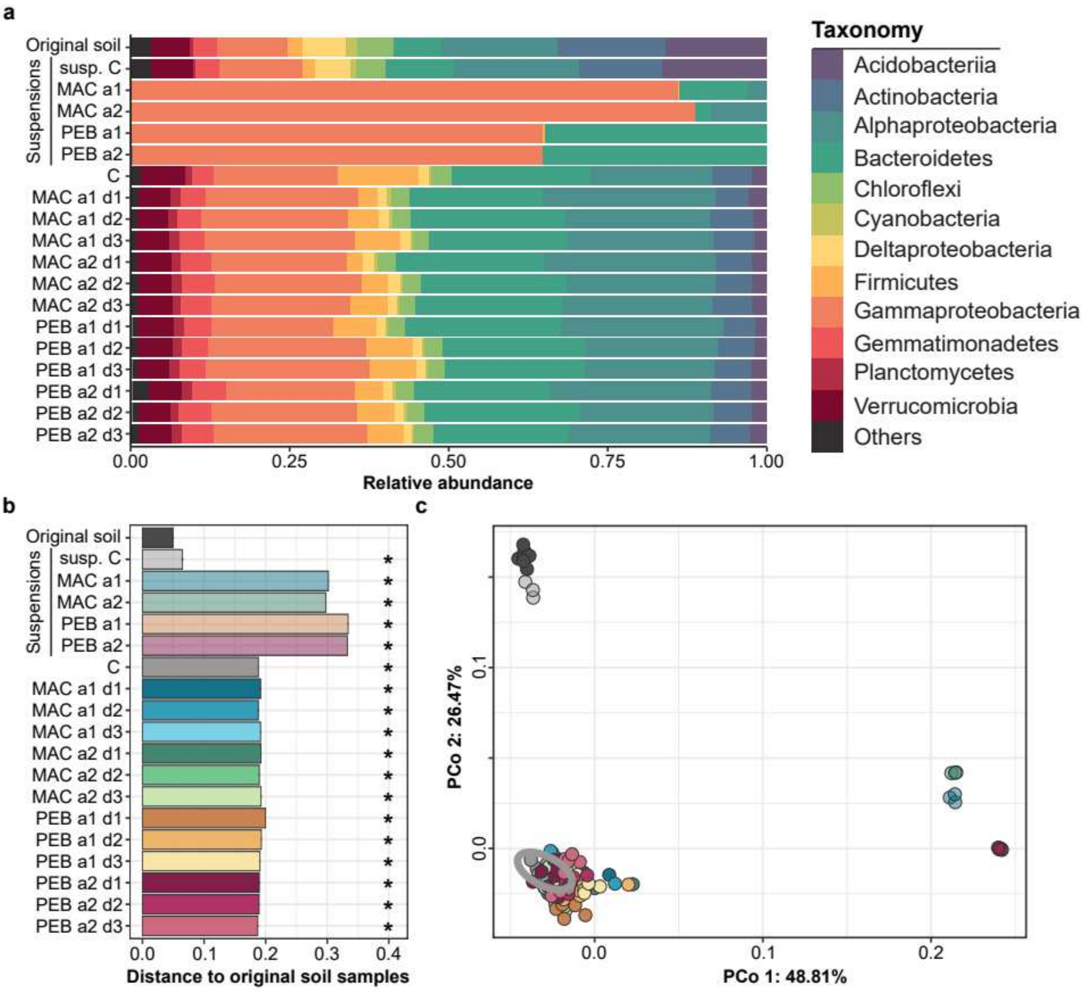

Supplementary Fig. S1: Composition and structure of the communities in the original soil, the non-treated control suspension, the manipulated suspensions, the non-coalesced control and the coalescence outcomes. (a) Relative abundances of the fourteen most abundant class of bacteria in the original soil, the non-treated control suspension, the manipulated suspensions, the non-coalesced control and the coalescence outcomes. (b) Weighted UniFrac distances between the original soil samples and either themselves (Original soil), the non-treated control suspension, the manipulated suspensions, the non-coalesced control or the coalescenced communities (mean ± s.e.). Asterisks indicate communities significantly different than the original soil (Welch's t-test p-value ≤ 0.05). (c) Principal coordinate analysis (PCoA) based on the weighted UniFrac distance matrix showing the original soil, the non-treated control suspension, the manipulated suspensions, the non-

coalesced control and the coalescence outcomes and the 95% joint confidence ellipse for the non-coalesced control samples. The dot colors correspond to the bar colors in b.

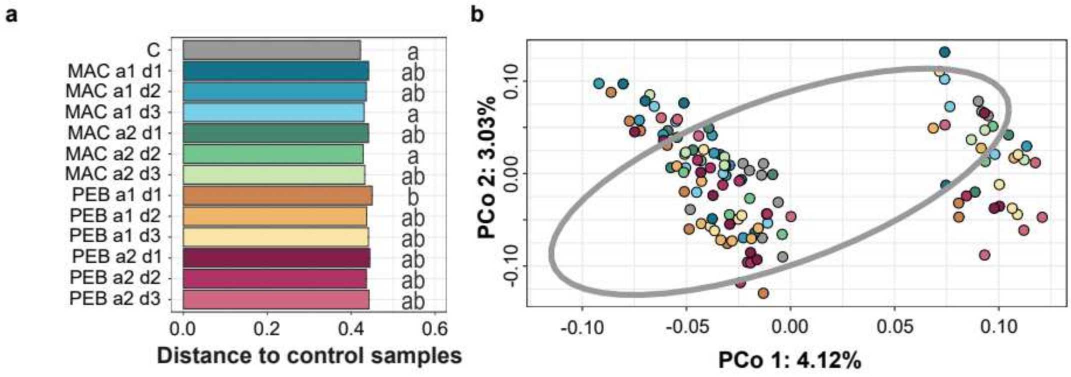

Supplementary Fig. S2: Unweighted UniFrac distances between the non-coalesced control and the coalesced communities. (a) Bars show the unweighted UniFrac distances between the non-coalesced control samples and either themselves (C) or the coalesced communities (mean ± s.e.). Letters indicate significantly different statistical groups (Adonis pairwise comparison, Benjamini-Hochberg corrected p-value ≤ 0.05). (b) Principal coordinate analysis (PCoA) based on the unweighted UniFrac distance matrix showing the non-coalesced control and the coalesced community samples and the 95% joint confidence ellipse for the non-coalesced control samples. The dot colors correspond to the bar colors in a.

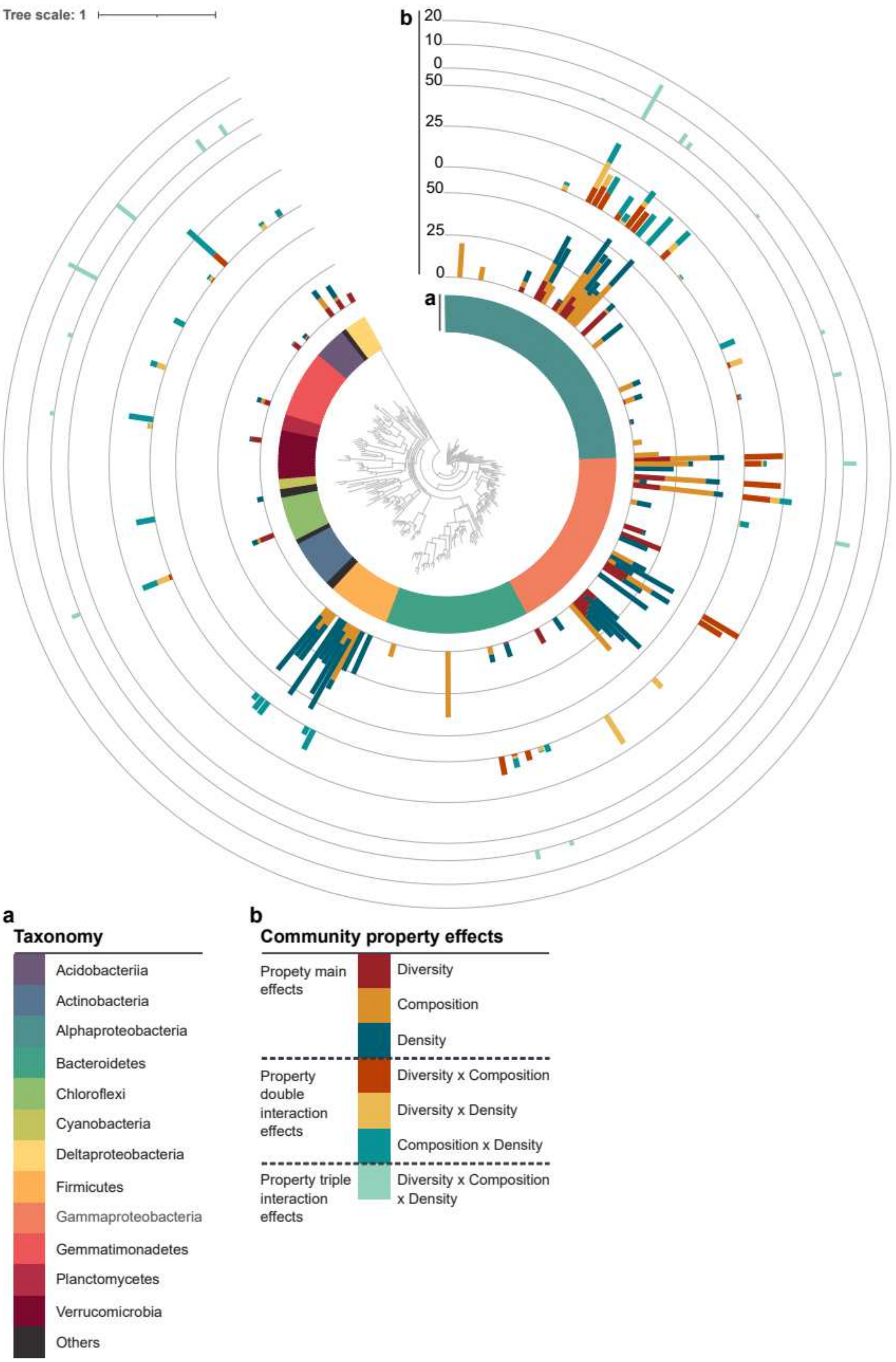

Supplementary Fig. S3: Community property effects on the 258 most abundant 16S rRNA OTUs' abundance. (a) The OTU class level is indicated by different colors on the innermost ring. (b) Bars

show the percentage of variance explained by each community property on the OTUs' relative abundances, as estimated using a generalized linear mixed model (Bonferroni adjusted p-value ≤ 0.05).

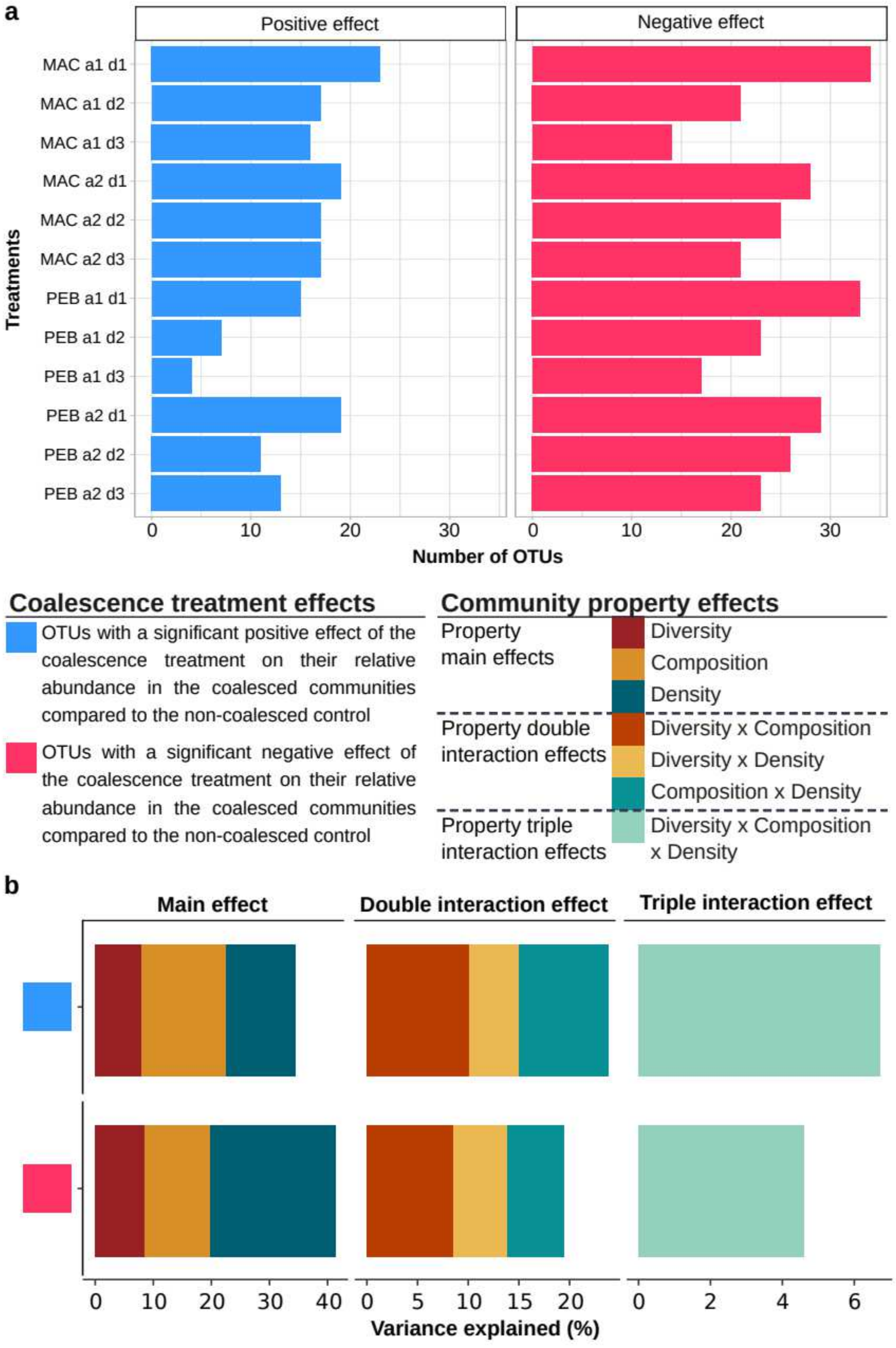

Supplementary Fig. S4: Effect of coalescence treatments and relative effect of community properties on the relative abundance of the most abundant OTUs. (a) Number of OTUs with either a significant

positive or negative effect of the coalescence treatment on their relative abundance in the coalesced communities compared to the non-coalesced control, as estimated using a generalized linear mixed model (Bonferroni adjusted p-value ≤ 0.05). (b) Bars show the percentage of variance explained by each community property on the relative abundances of OTUs either positively or negatively affected by coalescence treatments, as estimated using a generalized linear mixed model (Bonferroni adjusted p-value ≤ 0.05).

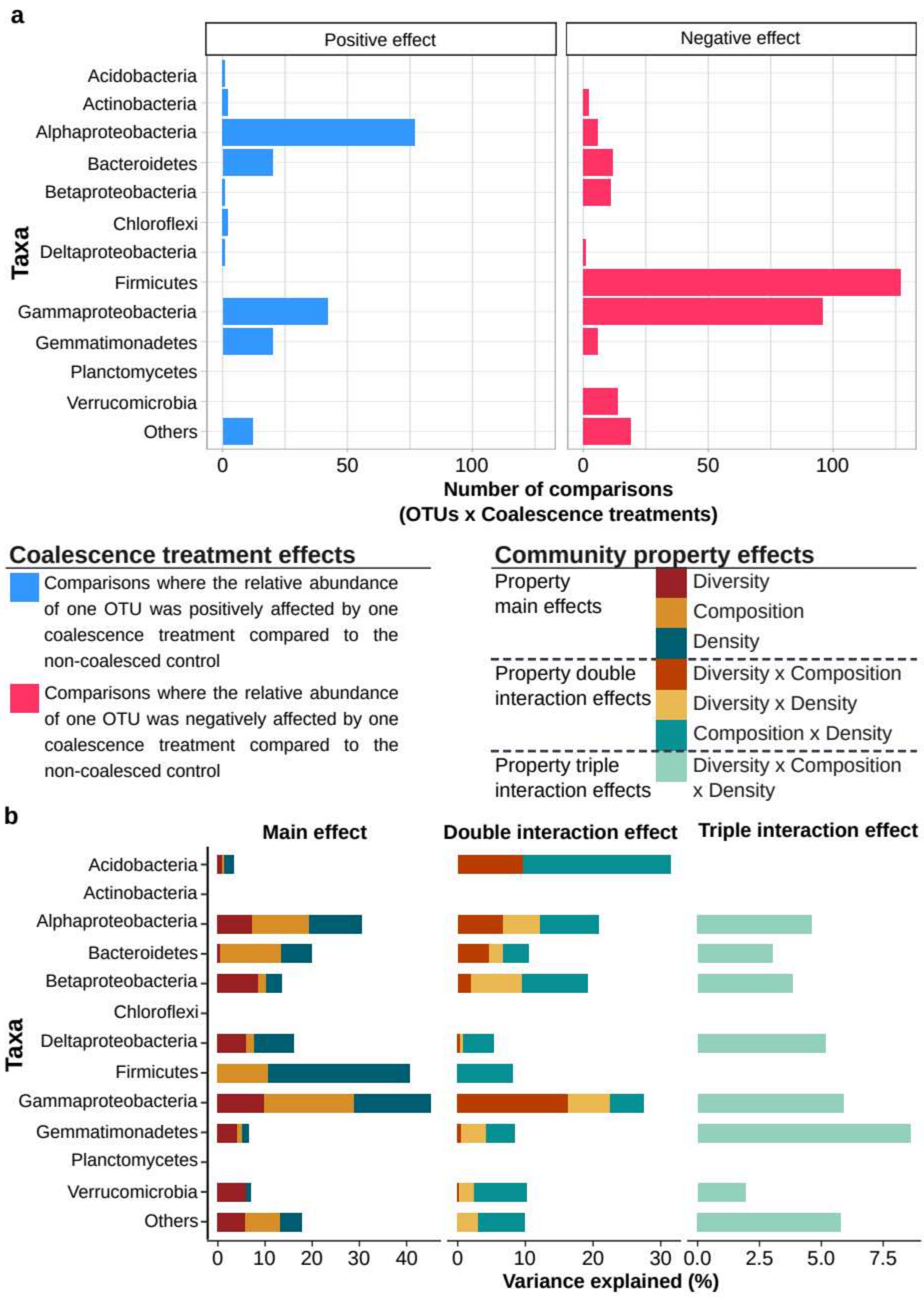

Supplementary Fig. S5: Effect of coalescence treatments and relative effect of community properties on the relative abundance of OTUs depending on their taxonomic affiliation. (a) Number of OTUs with either a significant positive or negative effect of the coalescence treatment on their relative abundance in the coalesced communities compared to the non-coalesced control, depending on

their taxonomic affiliation, as estimated using a generalized linear mixed model (Bonferroni adjusted p-value ≤ 0.05).(b) Bars show the percentage of variance explained by each community property on the relative abundances of OTUs depending on their taxonomic affiliation, as estimated using a generalized linear mixed model (Bonferroni adjusted p-value ≤ 0.05).

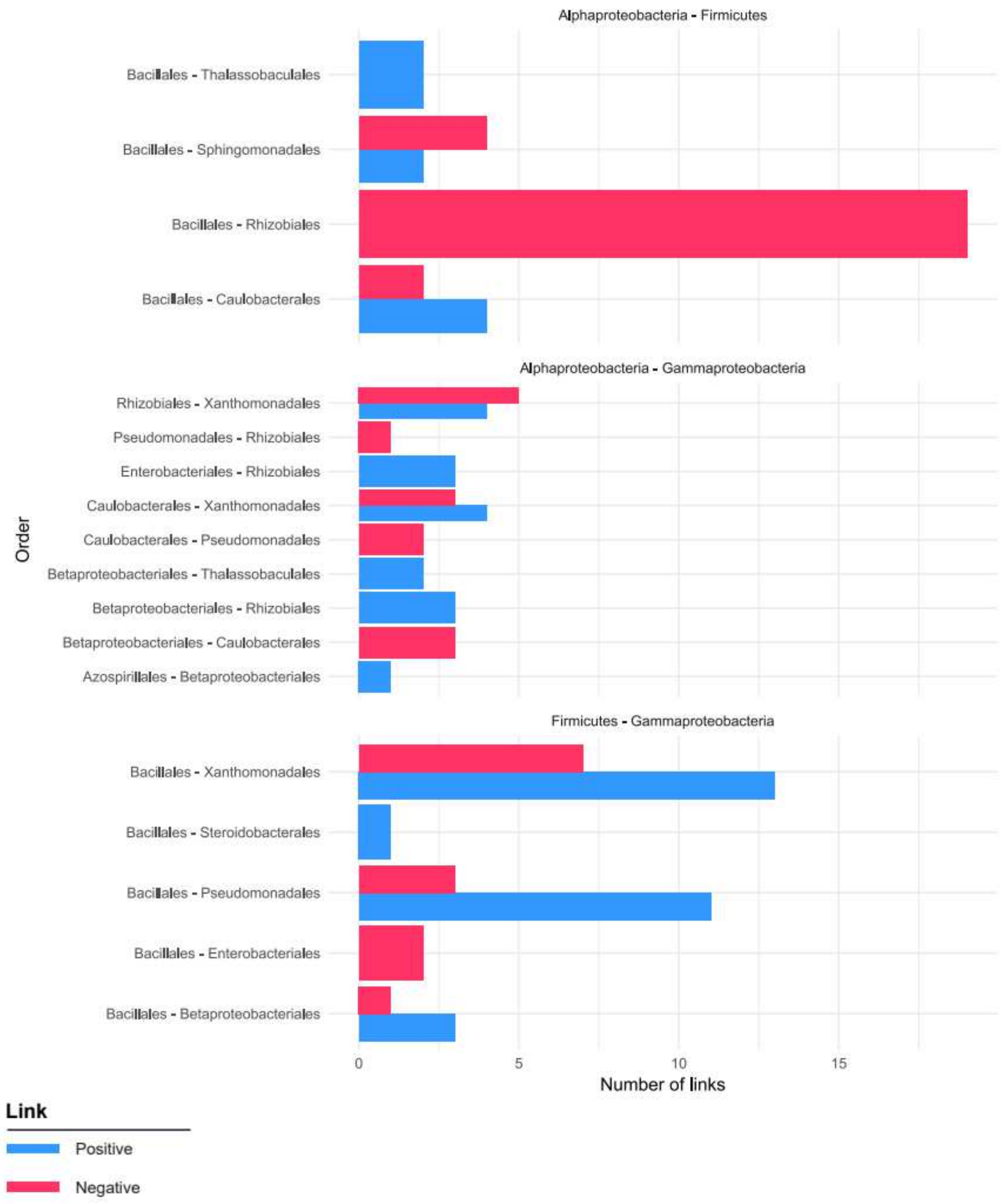

Supplementary Fig. S6: Number of network links between α-Proteobacteria, γ-Protebacteria and Firmicutes (order level).
